# Cognitive impairment in adolescent and adult-onset psychosis: a comparative study

**DOI:** 10.1186/s13034-024-00815-y

**Published:** 2024-09-28

**Authors:** TianHong Zhang, YanYan Wei, XiaoChen Tang, LiHua Xu, HuiRu Cui, YeGang Hu, HaiChun Liu, ZiXuan Wang, Tao Chen, ChunBo Li, JiJun Wang

**Affiliations:** 1 Shanghai Mental Health Center, Shanghai Engineering Research Center of Intelligent Psychological Evaluation and Intervention (20DZ2253800), Shanghai Key Laboratory of Psychotic Disorders (No.13dz2260500), Shanghai Jiaotong University School of Medicine, 600 Wanping Nan Road, Shanghai, 200030 China; 2 Department of Automation, Shanghai Jiao Tong University, Shanghai, 200240 China; 3 Shanghai Xinlianxin Psychological Counseling Center, Shanghai, China; 4 Big Data Research Lab, University of Waterloo, Waterloo, ON Canada; 5 Labor and Worklife Program, Harvard University, Massachusetts, USA; 6 Center for Excellence in Brain Science and Intelligence Technology (CEBSIT), Chinese Academy of Science, Beijing, PR China; 7 Institute of Psychology and Behavioral Science, Shanghai Jiao Tong University, Shanghai, PR China

**Keywords:** Psychotic disorders, Schizophrenia, Adolescent, Cognitive neuroscience, Early onset

## Abstract

**Background:**

Cognitive impairment presents in both adolescent-onset(ado-OP) and adult-onset psychosis(adu-OP). Age and neurodevelopmental factors likely contribute to cognitive differences. This study aimed to characterize cognitive functions in ado-OP compared to adu-OP in a clinical population with drug-naive first-episode psychosis(FEP).

**Methods:**

A total of 788 drug-naive patients with FEP and 774 sex- and age-matched healthy controls(HCs) were included. Participants were divided into four groups by whether they were under or over 21 years of age: adolescent-onset FEP(ado-FEP, *n* = 380), adult-onset FEP(adu-FEP, *n* = 408), adolescent HC(ado-HC, *n* = 334), and adult HC(adu-HC, *n* = 440). Comprehensive cognitive assessments were performed using the MATRICS Cognitive Consensus Battery(MCCB), covers six cognitive domains: speed of processing, attention/vigilance, working memory, verbal learning, visual learning, reasoning, and problem-solving. Data analyses were conducted using correlation analyses and binary logistic regression.

**Results:**

The patterns of cognitive domain differences between ado-FEP and adu-FEP were found to be similar to those between ado-HC and adu-HC, whereas cognitive impairments appeared to be more pronounced in patients with adu-OP than ado-OP. The mazes subtest had the maximum effect size(ES) in the FEP(ES = 0.37) and HC(ES = 0.30) groups when comparing the adolescent and adult groups. Cognitive subtests were mostly significantly correlated with negative symptoms, especially for adolescents with FEP, in which all the subtests were significantly correlated with negative symptoms in the ado-FEP group. Better performance in the domains of spatial cognition and problem-solving abilities was more likely in the ado-FEP group than in the adu-FEP group.

**Conclusions:**

These findings suggest cognitive differences between adolescents and adults but similar patterns of affected domains in HCs and patients with FEP. Therefore, the development of targeted cognitive interventions tailored to the specific needs of different age groups appears warranted.

## Introduction

Adolescent-onset psychosis (ado-OP) [1] is characterized by the manifestation of psychotic symptoms during adolescence. When psychosis emerges during adolescence, it presents unique challenges and considerations specific to the adolescent population. Ado-OP may exhibit a range of symptoms similar to those seen in adult-onset psychosis (adu-OP), including hallucinations, delusions, disorganized speech, and negative symptoms, such as social withdrawal and decreased motivation. However, the presence of these symptoms during a critical period of cognitive, emotional, and social development can have a profound impact on overall functioning and psychosocial adjustment [2, 3].

Studying ado-OP presents several challenges. Adolescence is a period of significant neurodevelopmental changes, making it difficult to disentangle the effects of psychosis from typical developmental processes [4]. Additionally, adolescents may have varying levels of cognitive and emotional maturity, which can complicate the assessment of symptoms and cognitive deficits [5]. Moreover, more negative symptoms [6] and prodromal symptoms [7], as well as different premorbid characteristics [8], have been observed in early-onset psychosis compared to adult-onset psychosis. Furthermore, ethical considerations arise when working with this vulnerable population, particularly in relation to consent and the potential impact of research participation on their mental health. Despite these challenges, research in this area is crucial for understanding the unique aspects of ado-OP and developing age-appropriate interventions.

Ado-OP is characterized by a more severe course of illness and poorer long-term outcomes than adu-OP [3, 9]. Cognitive impairment is a common feature and can manifest before the onset of psychotic symptoms [10, 11]. Research suggests that these cognitive deficits are present early in the course of illness [12, 13], especially during adolescence and childhood [14]. De la Serna et al. [15] found that patients with an earlier age of psychosis onset showed greater impairment in global cognition, executive functioning, and sustained attention. White et al. [16] reported that first-episode adolescent schizophrenia patients performed worse than adult patients on working memory, language, and motor function tasks, suggesting the onset of schizophrenia during adolescence may lead to a cessation in the development of specific cognitive domains. The causes of cognitive impairment in ado-OP are multifaceted and include neurodevelopmental abnormalities [17, 18], genetic factors [19], and altered brain connectivity [20, 21]. In addition, environmental factors [22] such as early life stress, substance abuse, and poor social support, can further impact cognitive functioning in individuals with ado-OP.

Understanding specific cognitive functioning in ado-OP is important for early detection, accurate diagnosis, and appropriate intervention strategies [23]. Identifying cognitive deficits early in the course of ado-OP can help clinicians develop targeted treatment plans that address these impairments and potentially improve long-term outcomes. For example, targeted cognitive remediation [24] programs and comprehensive treatment approaches that address cognitive deficits may help mitigate the impact of these impairments and improve the overall functional outcomes of individuals with ado-OP. Moreover, understanding the cognitive profiles of ado-OP can aid in distinguishing ado-OP from other psychiatric disorders, leading to more accurate diagnoses and personalized interventions.

Although cognitive deficits have been widely reported in patients with ado-OP, few studies have directly compared adolescent and adult patients with FEP [25] with well-matched controls. Previous studies had small sample sizes [2, 14], confounding effects of concomitant antipsychotic use [3], and participants with substance abuse [9, 26]. In this study, we aimed to perform a comprehensive analysis of cognitive functions in patients with first-episode psychosis (FEP) that onset in adolescence or adulthood. By evaluating a larger sample size, including both clinical and healthy controls (HCs) and carefully controlling for potential confounding factors, our study sought to provide a more robust understanding of the specific cognitive characteristics associated with ado-OP.

## Methods

### Subjects

Participants in the current study were recruited from ten psychiatric tertiary hospitals in China based on the National Key R&D Program of the Ministry of Science and Technology of China (2016YFC1306800) conducted between 2016 and 2021. This project aimed to explore behavioral and biological markers for the stage identification of psychosis and to develop treatments for early intervention. A total of 788 consecutive patients with FEP (males: 399; females: 389) in these hospitals and 774 well-matched HCs from local communities were enrolled. Participants must be under 35 years of age. Participants were included in the study with an established diagnosis of FEP, as identified by a certified psychiatrist in accordance with the Diagnostic and Statistical Manual of Mental Disorders, Fourth Edition, Text Revision (DSM-IV-TR). The diagnoses considered as psychotic disorders in this study included schizophrenia, schizoaffective disorder, schizophreniform disorder, brief psychotic disorder, and psychotic disorder not otherwise specified (NOS). To be eligible for inclusion, patients had to be within their first 2 years of experiencing psychotic symptoms, as determined by their first presentation to a clinical setting, and were required to have not received any prescribed antipsychotic medication prior to the study. The presence of frank psychotic symptoms, such as hallucinations, delusions, and disorganized thinking, was essential for inclusion in the study. However, FEP participants must be in a relatively stable condition, as judged by a clinician, with no significant risk of agitation or impulsivity, enabling them to complete clinical assessments and cognitive testing. They did not have any history of substance abuse or dependence according to the specific exclusion criteria.

The HC group was recruited from the communities surrounding the 10 centers participating in the study. Each center was responsible for recruiting sex-, age-, and education-matched participants from their respective cities. The recruitment process was carefully designed to ensure that the HCs were representative of the general population in terms of demographic factors, thereby allowing for meaningful comparisons with the FEP group. The inclusion criteria for the HC group were otherwise identical to those of the FEP group, with the primary exception being the absence of a psychotic disorder diagnosis. Exclusion Criteria for HC Group: (1) A history of any psychiatric disorder; (2) Current or previous use of psychiatric medications; (3) A history of substance abuse or dependence; (4) A first-degree relative with a history of psychosis; (5) Sensory impairments (e.g., visual or auditory) that could interfere with the completion of cognitive assessments; (6) Any physical health condition that could prevent the participant from completing the cognitive tests.

The project was led by the Shanghai Mental Health Center (SMHC), and all procedures involving human subjects/patients were approved by the Research Ethics Committee of SMHC (IRB2016-009). The relevant research ethics committees at different sites approved these studies. All participants provided written informed consent during recruitment. All procedures contributing to this work complied with the ethical standards of the relevant national and institutional committees on human experimentation and the 1975 Declaration of Helsinki, as revised in 2008.

### Symptomatic assessments

The clinical assessment was completed on the same day as enrollment. Face-to-face interviews were conducted using the Positive and Negative Syndrome Scale (PANSS) [27]. The PANSS consists of 30 items divided into three subscales: positive, negative, and general psychopathology. Each item is rated on a 7-point Likert scale (1 = absent to 7 = extreme). Structured clinical interviews were conducted with 23 senior psychiatrists who had completed the training required for this type of investigation. The inter-rater reliability for the DSM-IV-TR FEP diagnosis and PANSS ranged from 0.76 to 0.92 among the trained interviewers.

### Cognitive assessments

Cognitive testing was conducted when the patients’ clinical symptoms were relatively stable, with no significant risk of agitation or impulsivity. This ensured that the patients were in a suitable state to participate in the assessments effectively. The Chinese version of the Measurement and Treatment Research to Improve Cognition in Schizophrenia Consensus Cognitive Battery (MCCB) [28–30] was used for the cognitive assessments. The Chinese version of the MCCB included the following eight subtests: (1) Part A of the Trail-Making Test (Trail-Making A), (2) Symbol Coding of the Brief Assessment of Cognition in Schizophrenia (BACS symbol coding), (3) Category Fluency Test (category fluency), (4) Continuous Performance Test–Identical Pairs (CPT-IP), (5) Spatial Span of the Wechsler Memory Scale-III (WMS-3 spatial span), (6) Revised Hopkins Verbal Learning Test (HVLT-R), (7) Revised Brief Visuospatial Memory Test (BVMT-R), and (8) Neuropsychological Assessment Battery: Mazes (NAB mazes). Test-retest reliability in a previous Chinese psychosis sample ranged from 0.73 to 0.94 [30]. The MCCB covers six cognitive domains: speed of processing (Trail-Making A, BACS symbol coding, and category fluency), attention/vigilance (CPT-IP), working memory (WMS-3 spatial span), verbal learning (HVLT-R), visual learning (BVMT-R), reasoning, and problem-solving (NAB mazes).

The cognitive assessments were performed by trained researchers at each center. At the start of the project, all cognitive assessors underwent standardized training on the MCCB. The training was conducted by recognized experts in MCCB testing in China. The training included practice with no fewer than 10 test cases. Only those assessors who successfully passed an on-site evaluation by the cognitive assessment trainer were certified to conduct the assessments.

### Data analysis

SPSS for Windows (version 20.0; IBM, Armonk, NY, USA) and the R statistical software package (version 4.1.2; R Foundation for Statistical Computing, Vienna, Austria) were both utilized for the data analysis. Participants were divided into four groups based on adolescent and adult groups according to their age: 21 years or younger and older than 21 years [31], including adolescent-onset FEP (ado-FEP), adult-onset FEP (adu-FEP), adolescent HC (ado-HC), and adult HC (adu-HC). To determine the differences in performance on neurocognitive subtests in the MCCB, we calculated z-scores for the ado- and adu-FEP groups based on the means and standard deviations (SD) of the ado- and adu-HC participants and compared them using an independent t-test analysis of variance. The effect size (ES) was evaluated as η^2^ = 0.01 (small), η^2^ = 0.06 (medium), and η^2^ = 0.14 (large). Spearman’s correlation analysis was conducted to explore the association between the severity of clinical symptoms and cognitive functions. Statistical comparisons between the ado- and adu-FEP groups were conducted using the package cocor [32] in the R programming language (http://comparingcronbachalphas.org). Binary logistic regression was used to determine adjusted associations of cognitive performance between the ado-FEP, adu-FEP, ado-HC, and adu-HC groups. A backward selection procedure was used to find the most parsimonious model, and the Hosmer–Lemeshaw goodness of fit test was used to determine the model fitness. The odds ratios (OR) and 95% confidence intervals (CIs) for covariates were reported.

## Results

The demographic and clinical characteristics of the 788 FEP and 774 HC participants are shown in Table 1. The mean age was not significantly different between the FEP (22.7 ± 6.3) and HC (22.4 ± 4.9) groups. The age range for the entire sample, including both FEP and HC participants, was 10 to 35 years (subgroup age ranges: ado-FEP, 11 to 21 years; adu-FEP, 22 to 35 years; ado-HC, 10 to 21 years; adu-HC, 22 to 35 years). The ado-FEP group had a higher proportion of males and a lower educational level than the adu-FEP group. Additionally, the adu-FEP group had higher positive symptom scores compared to the ado-FEP group.

**Table 1:** Demographic and clinical characteristics and comparisons among HC, FEP, ado-FEP, and adu-FEP groups

Neurocognitive variables	HC		FEP		Ado-FEP		Adu-FEP		Comparisons	
									t/χ^2^	*p*
Cases [n, %]	774		788		380	48.22%	408	51.78%	-	-
Age (years) [mean, SD.]	22.40	4.880	22.71	6.262	17.16	2.303	27.89	3.900	-46.604	< 0.001
Male [n, %]	366	47.3%	399	50.6%	207	54.47%	173	45.53%	4.328	0.037
Female [n, %]	408	52.7%	389	49.4%	192	47.06%	216	52.94%		
Education(years) [mean, SD.]	14.32	3.097	11.60	3.053	10.69	2.307	12.46	3.398	-8.507	< 0.001
Father Education(years) [mean, SD.]	11.05	3.396	9.68	3.546	9.70	3.498	9.65	3.621	0.161	0.872
Mother Education(years) [mean, SD.]	10.29	3.718	8.71	3.732	8.73	3.427	8.69	4.137	0.137	0.891
Family history (none) [n, %]	774	100%	598	75.89%	279	73.42%	319	78.19%	2.447	0.294
Family history (low-risk) [n, %]	0	0%	103	13.07%	55	14.47%	48	11.76%		
Family history (high-risk) [n, %]	0	0%	87	11.04%	46	12.11%	41	10.05%		
Positive symptoms [mean, SD.]	-	-	21.56	6.073	20.74	6.324	22.31	5.735	-3.658	< 0.001
Negative symptoms [mean, SD.]	-	-	18.13	7.268	18.23	7.524	18.03	7.029	0.390	0.696
General symptoms [mean, SD.]	-	-	39.72	8.401	39.63	8.492	39.79	8.325	-0.267	0.790
PANSS total score[mean, SD.]	-	-	79.39	16.925	78.59	17.395	80.14	16.462	-1.288	0.198

*SD * standard deviation, None family history: having no family members with mental disorders; Low-risk family history: a first-degree relative with non-psychotic disorders; High-risk family history: having at least one first-degree relative with psychosis

### Comparative analyses

**Fig. 1:**
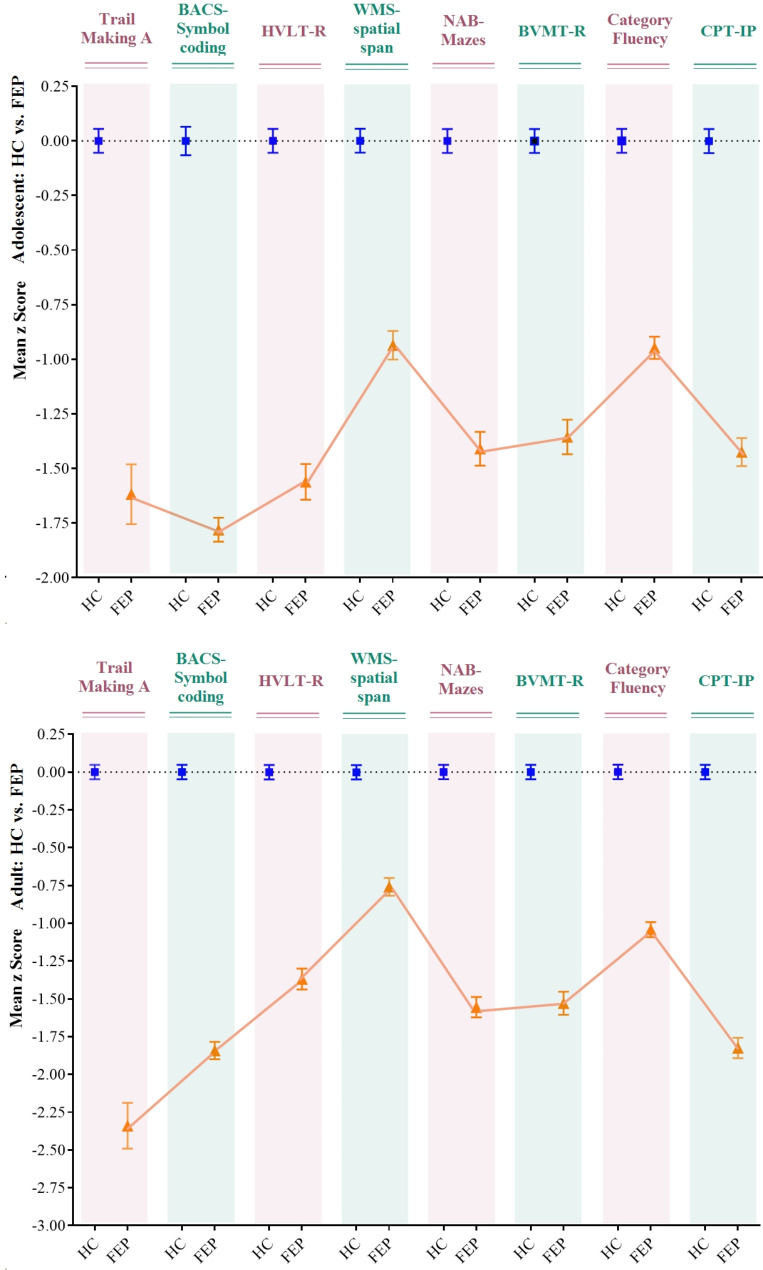
Neuropsychological comparisons between first-episode psychosis (FEP) and healthy controls (HC), stratified by adolescent and adult groups. z-scores for the ado- and adu-FEP groups based on the means and standard deviations (SD) of ado- and adu-HC participants. BACS, Brief Assessment of Cognition in Schizophrenia symbol coding; BVMT-R, Brief Visuospatial Memory Test–Revised; CPT-IP, Continuous Performance Test–Identical Pairs; HVLT-R, Hopkins Verbal Learning Test–Revised; NAB, Neuropsychological Assessment Battery mazes; WMS-3, Wechsler Memory Scale–Third Edition spatial span

In the HC groups, the mean scores of the NAB mazes (*t* = 4.060, *p* < 0.001) and WMS-3 spatial span (*t* = 3.608, *p* < 0.001) were significantly higher, and those of CPT-IP (*t*=-4.683, *p* < 0.001) and category fluency (*t*=-2.879, *p* = 0.004) were significantly lower in the adolescent groups than in the adult groups (Fig. 1), (Table 2). In the patients with FEP, the mean scores of the NAB mazes (*t* = 5.223, *p* < 0.001), BVMT-R (*t* = 3.107, *p* = 0.002), and BACS symbol coding (*t* = 3.062, *p* = 0.002) were significantly higher in the adolescent group than in the adult group.

**Table 2:** Neuropsychological performance profile and comparisons among ado-HC, adu-HC, ado-FEP, and adu-FEP groups

Neurocognitive variables	Ado-HC		Adu-HC		Comparisons		Ado-FEP		Adu-FEP		Comparisons	
	Mean	SD.	Mean	SD.	t	*p*	Mean	SD.	Mean	SD.	t	*p*
Trail Making A	29.01	11.319	28.85	9.919	0.207	0.836	47.33	30.285	52.06	30.319	-2.193	0.029
BACS symbol coding	65.46	9.892	64.32	10.396	1.544	0.123	47.85	12.543	45.16	12.073	3.062	**0.002**
HVLT-R	26.97	3.962	26.29	4.316	2.268	0.024	20.78	6.323	20.38	6.009	0.920	0.358
WMS-3 spatial span	17.15	2.887	16.38	3.029	3.608	**< 0.001**	14.45	3.674	14.08	3.581	1.418	0.157
NAB mazes	20.19	4.685	18.76	4.979	4.060	**< 0.001**	13.59	7.082	11.01	6.709	5.223	**< 0.001**
BVMT-R	28.88	5.323	27.97	5.311	2.357	0.019	21.66	8.178	19.85	8.199	3.107	**0.002**
Category Fluency	22.93	5.665	24.12	5.686	-2.879	**0.004**	17.56	5.607	18.19	5.721	-1.548	0.122
CPT-IP	2.78	0.690	3.00	0.620	-4.683	**< 0.001**	1.79	0.829	1.87	0.833	-1.214	0.225

Corrected *p* by controlling the family-wise error, at the 0.006 (*p* < 0.05/8[MCCB subtests] was significant) level using a Bonferroni correction. Abbreviations: BACS, Brief Assessment of Cognition in Schizophrenia symbol coding; BVMT-R, Brief Visuospatial Memory Test–Revised; CPT-IP, Continuous Performance Test–Identical Pairs; HVLT-R, Hopkins Verbal Learning Test–Revised; NAB, Neuropsychological Assessment Battery mazes; WMS-3, Wechsler Memory Scale–Third Edition spatial span

### ES analyses

**Fig. 2:**
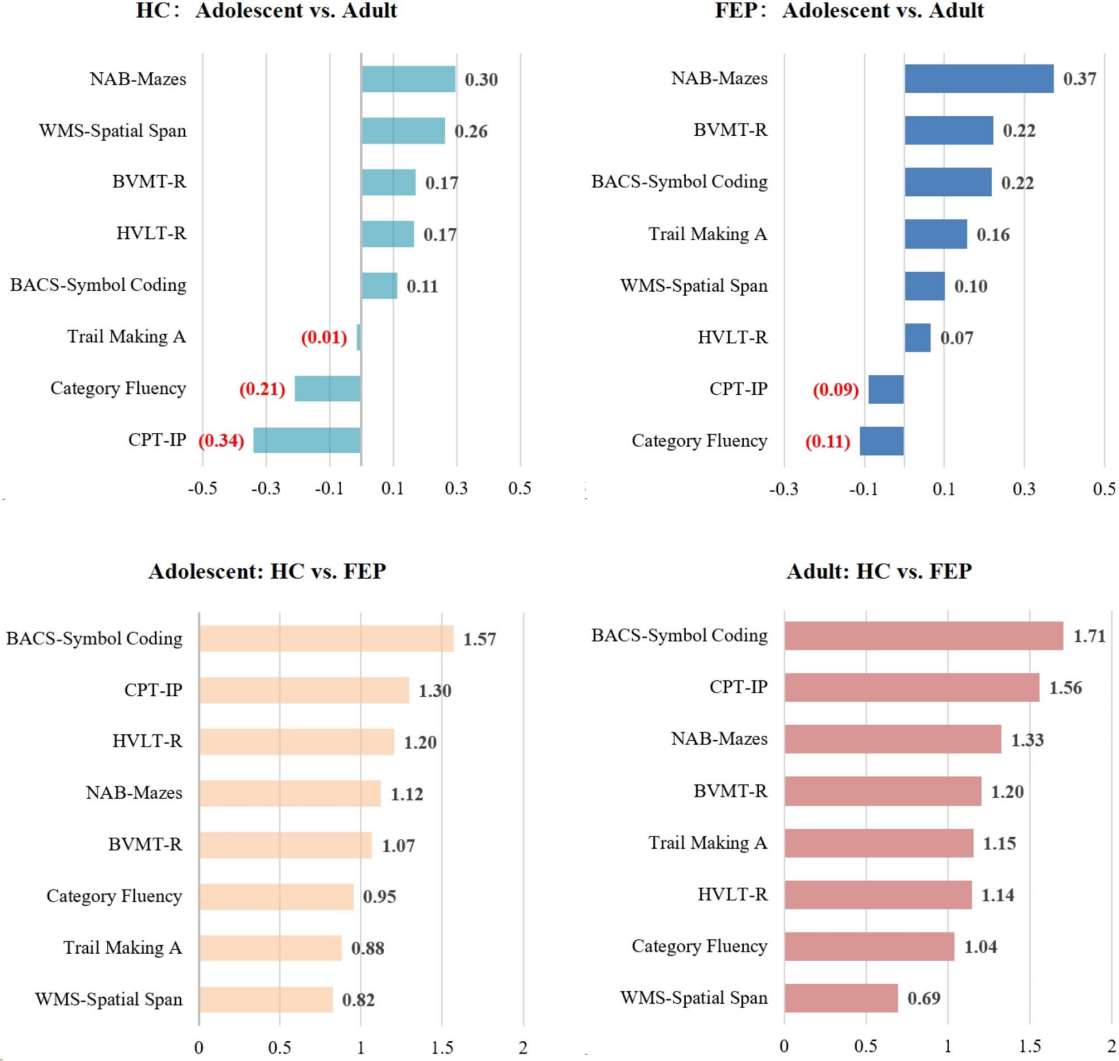
Effect sizes (Cohen *d*) for cognitive comparisons among healthy controls (HC) groups included ado-HC and adu-HC, and first-episode psychosis (FEP) groups included ado-FEP and adu-FEP groups Note: BACS, Brief Assessment of Cognition in Schizophrenia symbol coding; BVMT-R, Brief Visuospatial Memory Test–Revised; CPT-IP, Continuous Performance Test–Identical Pairs; HVLT-R, Hopkins Verbal Learning Test–Revised; NAB, Neuropsychological Assessment Battery mazes; WMS-3, Wechsler Memory Scale–Third Edition spatial span

In comparing the adolescent and adult groups, the NAB mazes test had the maximum ES in the FEP and HC groups (Fig. 2). Specifically, adolescent participants performed better in the NAB mazes test but worse in the CPT-IP and BACS category fluency tests than adult participants. In comparing the HC and FEP groups, BACS category fluency and CPT-IP—the top two tests—had the maximum ES in the adolescent and adult groups.

### Correlation analyses

Except for the NAB mazes test, all MCCB subtests were insignificantly correlated with positive symptoms, and the only significant correlation with general symptoms was found in the category fluency in the overall FEP group (Table 3). Meanwhile, the cognitive subtests were mostly significantly correlated with negative symptoms, particularly in adolescents with FEP. All MCCB subtests were significantly correlated with negative symptoms in the ado-FEP group. In the adu-FEP group, the Trail-Making A, BACS symbol coding, NAB maze, category fluency, and CPT-IP subtests were significantly correlated with negative symptoms. The HVLT-R subtest score showed more significant correlations with negative and general symptoms in the ado-FEP group than in the adu-FEP group.

**Table 3:** Correlations between clinical symptoms and neurocognitive performances, stratified by ado-FEP and adu-FEP groups

Neurocognitive variables	Overall		Ado-FEP		Adu-FEP		Comparisons	
	*r*	*p*	*r*	*p*	*r*	*p*	Fisher’s z	*p*
Positive symptoms								
Trail Making A	0.051	0.156	0.016	0.756	0.056	0.255	-0.560	0.576
BACS symbol coding	-0.076*	0.034	-0.070	0.173	-0.058	0.242	-0.168	0.866
HVLT-R	0.006	0.864	-0.038	0.463	0.057	0.255	-1.329	0.184
WMS-3 spatial span	-0.061	0.090	-0.066	0.200	-0.048	0.333	-0.252	0.801
NAB mazes	-0.133**	< 0.001	-0.113*	0.028	-0.112*	0.024	-0.014	0.989
BVMT-R	-0.033	0.360	-0.055	0.292	0.007	0.887	-0.867	0.386
Category Fluency	-0.054	0.132	-0.045	0.385	-0.075	0.134	0.421	0.674
CPT-IP	-0.068	0.066	-0.108*	0.043	-0.042	0.410	-0.928	0.354
Negative symptoms								
Trail Making A	0.173**	< 0.001	0.178**	< 0.001	0.177**	< 0.001	0.014	0.989
BACS symbol coding	-0.217**	< 0.001	-0.281**	< 0.001	-0.159**	0.001	-1.794	0.073
HVLT-R	-0.099**	0.006	-0.170**	0.001	-0.031	0.539	-1.965	**0.049**
WMS-3 spatial span	-0.106**	0.003	-0.149**	0.004	-0.056	0.261	-1.314	0.189
NAB mazes	-0.117**	0.001	-0.115*	0.026	-0.127*	0.011	0.170	0.865
BVMT-R	-0.068	0.056	-0.135**	0.009	-0.010	0.841	-1.758	0.079
Category Fluency	-0.142**	< 0.001	-0.149**	0.004	-0.136**	0.006	-0.185	0.853
CPT-IP	-0.177**	< 0.001	-0.188**	< 0.001	-0.170**	0.001	-0.260	0.795
General symptoms								
Trail making A	-0.014	0.696	-0.013	0.806	-0.009	0.852	-0.056	0.955
BACS symbol coding	-0.064	0.071	-0.112*	0.030	-0.018	0.717	-1.320	0.187
HVLT-R	-0.026	0.470	-0.101*	0.049	0.048	0.336	-2.087	**0.037**
WMS-3 spatial span	-0.002	0.962	-0.064	0.217	0.064	0.198	-1.791	0.073
NAB mazes	-0.001	0.987	-0.016	0.752	0.013	0.799	-0.405	0.685
BVMT-R	-0.004	0.909	-0.066	0.204	0.050	0.309	-1.623	0.105
Category Fluency	-0.092**	0.010	-0.080	0.121	-0.104*	0.035	0.338	0.735
CPT-IP	-0.057	0.122	-0.102	0.058	-0.017	0.733	-1.193	0.233

*BACS* Brief assessment of cognition in schizophrenia symbol coding, *BVMT-R* Brief visuospatial memory test–revised, *CPT-IP* Continuous performance test–identical Pairs, *HVLT-R* Hopkins verbal learning test–revised *NAB* Neuropsychological assessment battery mazes, *WMS-3* Wechsler Memory Scale–Third Edition spatial span. Statistical comparisons between correlations were conducted by the Cocor package.

* *p* < 0.05; ** *p* < 0.01

### Logistic regression analyses

According to the binary logistic regression analysis, poorer performance in the category fluency and CPT-IP tests and better performance in the BACS symbol coding, NAB mazes, and BVMT-R were more likely in patients with adolescent-onset than adult-onset FEP. A similar pattern was found in the comparison of the adolescent and adult HC groups (Table 4). When stratified by adolescents and adults, BACS symbol coding and CPT-IP were the top two significant discriminators between the FEP and HC groups.

**Table 4:** Binary logistic regression analysis of neurocognitive functions among ado-FEP and adu-FEP, ado-HC and adu-HC groups

Variables	Analysis						
	Beta	S.E.	OR	95%CI for OR		Wald	*P*
Ado-FEP vs. Adu-FEP (Classification accuracy: 63.8%) (Hosmer–Lemeshaw test: 11.163(0.193))							
BACS symbol coding	-0.022	0.009	0.978	0.962	0.995	6.432	0.011
NAB mazes	-0.059	0.014	0.943	0.918	0.969	18.319	< 0.001
BVMT-R	-0.040	0.012	0.960	0.938	0.983	11.385	0.001
Category fluency	0.037	0.016	1.037	1.006	1.070	5.530	0.019
CPT-IP	0.577	0.122	1.780	1.403	2.259	22.523	< 0.001
Ado-HC vs. Adu-HC (Classification accuracy: 65.7%) (Hosmer–Lemeshaw test: 6.752(0.564))							
BACS symbol coding	-0.023	0.009	0.977	0.960	0.994	6.844	0.009
HVLT-R	-0.062	0.021	0.940	0.902	0.980	8.522	0.004
WMS-3 spatial span	-0.081	0.029	0.923	0.871	0.977	7.521	0.006
NAB mazes	-0.062	0.018	0.940	0.907	0.975	11.340	0.001
Category fluency	0.059	0.015	1.060	1.029	1.093	14.363	< 0.001
CPT-IP	0.855	0.138	2.352	1.795	3.082	38.478	< 0.001
Ado-FEP vs. Ado-HC (Classification accuracy: 81.8%) (Hosmer–Lemeshaw test: 9.130(0.331))							
BACS symbol coding	-0.107	0.014	0.898	0.875	0.923	61.738	< 0.001
HVLT-R	-0.072	0.024	0.930	0.887	0.976	8.731	0.003
NAB mazes	-0.047	0.021	0.954	0.916	0.994	5.124	0.024
Category fluency	-0.055	0.022	0.947	0.907	0.988	6.317	0.012
CPT-IP	-0.614	0.163	0.541	0.393	0.746	14.097	0.001
Adu-FEP vs. Adu-HC (Classification accuracy: 84.6%) (Hosmer–Lemeshaw test: 21.609(0.006))							
Trail making A	0.033	0.010	1.034	1.014	1.054	11.160	0.001
BACS symbol coding	-0.083	0.012	0.920	0.898	0.943	44.766	< 0.001
HVLT-R	-0.059	0.023	0.943	0.901	0.987	6.470	0.011
NAB mazes	-0.064	0.020	0.938	0.902	0.975	10.658	0.001
CPT-IP	-1.066	0.171	0.344	0.246	0.482	38.741	< 0.001

Beta is the regression coefficient. S.E. is the standard error. 95% CI is the estimated 95% confidence interval for the corresponding parameter. OR is the standardized regression coefficient. Hosmer–Lemeshaw goodness of fit test: ***χ***^***2***^ (*p*); BACS, Brief Assessment of Cognition in Schizophrenia symbol coding; BVMT-R, Brief Visuospatial Memory Test–Revised; CPT-IP, Continuous Performance Test–Identical Pairs; HVLT-R, Hopkins Verbal Learning Test–Revised; NAB, Neuropsychological Assessment Battery mazes; WMS-3, Wechsler Memory Scale–Third Edition spatial span

## Disscussion

This study is based on large-scale controlled research and has identified some valuable findings. First, the patterns of cognitive domain differences between adolescent and adult-onset psychosis were found to be similar to the patterns of cognitive domain differences between adolescent and adult HCs. Second, upon visual inspection, it appears that cognitive impairments are more pronounced in adult-onset patients than in adolescent-onset patients. Third, there is a close relationship between negative symptoms and cognitive functioning. Lastly, adolescents tended to outperform adults in specific cognitive tests, such as the NAB maze and BVMT-R tests in both the FEP and HC groups.

The similarities in cognitive function differences between adolescents and adults in both psychosis and HC groups may be attributed to neurodevelopmental factors, which play a significant role in cognitive differences [33, 34], During adolescence and early adulthood, the brain undergoes substantial structural and functional changes, including synaptic pruning [35], myelination [36], and refinement of neural circuits [34]. These developmental processes are closely linked to cognitive functions, such as attention, memory, executive functions, and social cognition. In FEP patients, disruptions in these neurodevelopmental processes may lead to similar patterns of cognitive impairment in both adolescents and adults [37, 38].

Contrary to previous findings suggesting greater cognitive impairments in ado-OP patients [14, 39], this study found more pronounced impairments in adu-OP patients. This could be due to the greater neuroplasticity of the adolescent brain, which may offer some resilience against the cognitive impairments associated with psychosis [40]. Additionally, psychosocial factors unique to adolescence, such as strong social support systems and active educational engagement [41], may contribute to better cognitive functioning and offset some of the impairments associated with psychosis.

The NAB maze and BVMT-R tests, which assess spatial and problem-solving abilities, showed better performance in adolescents than adults. This finding aligns with research by Nitzburg et al. [42], which showed that visual memory domains and problem-solving abilities were better in adolescents (17–20 years) than in adults (20–23 years). Adolescents’ higher levels of neuroplasticity and learning capacity may lead to better performance on these tests [43], as they can adapt more quickly and use more flexible strategies. In addition, adolescents may have had more recent exposure to maze-like tasks or video games [44], which could enhance their familiarity with spatial navigation tasks.

Although this study included a large sample of clinical and control participants and minimized the influence of medications, several limitations should be acknowledged. The use of a cross-sectional design limited our ability to establish causal relationships or determine the trajectory of cognitive impairment over time. Longitudinal studies should provide more insight into the developmental patterns of cognitive deficits in different age groups. The intelligence quotient (IQ) is an important factor to consider when examining cognitive differences [45, 46]. The absence of IQ testing in this study prevented us from accounting for individual variations in baseline cognitive abilities, which could have impacted the interpretation of the results. The duration of untreated psychosis [47] or prodromal symptoms [48, 49] is a crucial variable in understanding the impact of early intervention on cognitive outcomes. The absence of these data limits our understanding of its potential influence on cognitive impairment. To address these limitations and further enhance our understanding of cognitive impairments in patients with adu-OP and adu-OP, longitudinal studies [50] that follow individuals from adolescence into adulthood should be conducted. Such studies would allow for a more comprehensive evaluation of cognitive trajectories and the identification of potential critical periods for intervention.

## Conclusion

This study demonstrates that cognitive differences between patients with ado-OP and adu-OP and HCs exhibit similar patterns across cognitive domains. In addition, our results suggest that cognitive impairments may be more pronounced in patients with adu-OP. Future research should focus on developing targeted cognitive interventions tailored to the specific needs of different age groups. Longitudinal studies are needed to better understand the trajectory of cognitive impairment and its impact on functional outcomes over time.

## Data Availability

Data generated during this study are not publicly available because participants did not agree for their data to be shared publicly. Individual deidentified, anonymized data are available from the authors upon reasonable request.

## Abbreviations

Ado-OP: Adolescent-onset
Ado-HC: Adolescent healthy control
Ado-FEP: Adolescent-onset first-episode psychosis
Adu-OP: Adult-onset psychosis
Adu-HC: Adult healthy control
Adu-FEP: Adult-onset first-episode psychosis
BACS: Brief assessment ofcCognition in schizophrenia
BVMT-R: Revised brief visuospatial memory test
CIs: Confidence intervals
CPT-IP: Continuous performance test–identical pairs
DSM-IV-TR: Diagnostic and statistical manual of mental disorders, fourth edition, text revision
ES: Effect size
FEP: First-episode psychosis
HCs: Healthy controls
HVLT-R: Revised Hopkins verbal learning test
MCCB: Measurement and treatment research to improve cognition in schizophrenia consensus cognitive battery
NAB: Neuropsychological assessment battery
OR: Odds ratios
PANSS: Positive and negative syndrome scale
SMHC: Shanghai Mental Health Center
Trail-Making A: Part A of the trail-making test
WMS-3: Wechsler memory scale-III
